# Investigation the biological activities and the metabolite profiles of endophytic fungi isolated from *Gundelia tournefortii* L.

**DOI:** 10.1038/s41598-024-57222-8

**Published:** 2024-03-25

**Authors:** Mostafa Ebadi, Fatemeh Ahmadi, Hossein Tahmouresi, Mohammad Pazhang, Saeed Mollaei

**Affiliations:** 1 Department of Biology, Faculty of Basic Sciences, Azarbaijan Shahid Madani University, Tabriz, Iran; 2 Department of Chemistry, Faculty of Basic Sciences, Azarbaijan Shahid Madani University, Tabriz, Iran

**Keywords:** Antioxidant, Enzyme, Fatty acid, Phenolic acid, Phylogeny, Microbiology, Plant sciences

## Abstract

Endophytic fungi are microorganisms that are considered as a potential source of natural compounds, and can be applied in various industries. The aims of this research were molecular identification of endophytic fungi isolated from the *Gundelia tournefortii* stems, and investigation their biological activities as well as phenolic and fatty acid profile. Surface sterilized stems of *G. tournefortii* were placed on potato dextrose agar (PDA) to isolate the fungal endophytes. Genomic DNA was extracted by CTAB method, and PCR amplification was performed by ITS 1 and ITS 4 as primers. The enzyme production of endophytic fungi was determined based on the formation of a clear zone that appeared around the colonies of fungus. The anti-oxidant activity was evaluated by measuring the amount of free radicals DPPH. Also, the total phenol and flavonoid contents were measured obtained by Folin-Ciocalteu and aluminum chloride colorimetric methods, respectively. Moreover, the separation and identification of phenolic acids and fatty acids were done by HPLC and GC, respectively. Phylogenetic analysis was done based on the Internal Transcribed Spacer (ITS) region, and five isolates were identified as following: *Aspergillus niger*, *Penicillium glabrum*, *Alternaria alternata*, *A. tenuissima*, and *Mucor circinelloides*. Evaluation of the enzymatic properties showed that *P. gabrum* (31 ± 1.9 mm), and *A. niger* (23 ± 1.7) had more ability for producing pectinase and cellulase. The anti-oxidant activity of isolates showed that *A. alternata* extract (IC_50_ = 471 ± 29 µg/mL) had the highest anti-oxidant properties, followed by *A. tenuissima* extract (IC_50_ = 512 ± 19 µg/mL). Also, the extract of *A. alternata* had the greatest amount of total phenols and flavonoids contents (8.2 ± 0.4 mg GAL/g and 2.3 ± 0.3 mg QE/g, respectively). The quantification analysis of phenolic acid showed that rosmarinic acid, para-coumaric acid, and meta-coumaric acid (42.02 ± 1.31, 7.53 ± 0.19, 5.41 ± 0.21 mg/g, respectively) were the main phenolic acids in the studied fungi. The analysis of fatty acids confirmed that, in all fungi, the main fatty acids were stearic acid (27.9–35.2%), oleic acid (11.3–17.3%), palmitic acid (16.9–23.2%), linoleic acid (5.8–11.6%), and caprylic acid (6.3–10.9%). Our finding showed that endophytic fungi are a source of bioactive compounds, which could be used in various industries. This is the first report of endophytic fungi associated with *G. tournefortii*, which provides knowledge on their future use on biotechnological processes.

## Introduction

Endophytic fungi are endosymbionts that live inside medicinal herbs tissues without causing disease. They protect the plant against biotic and abiotic stresses by different mechanisms^1,2^. They can independently synthesize secondary metabolites similar to those made by the host plants. The production of active compounds by them is thought to occur through coevolution or genetic transfer of the host plant to endophytes^3^.

Nowadays, the isolation and extraction of bioactive compounds from endophytic fungi are important. The studies have revealed the ability of endophytic fungi for producing many secondary metabolites such as phenols, quinones, xanthones, isocoumarins, terpenoids, alkaloids, and steroids that work as anti-cancer, anti-viral, anti-bacterial, insecticidal, anti-inflammatory, anti-fungal, anti-diabetic, anti-oxidant, and etc^4–6^.

Phenols are one of the bioactive compounds that can be extracted from endophytic fungi. In 2014, Huang et al.^7^ could identify kaempferol from *Mucor fragilis*, which was isolated from *Sinopodophyllum hexandrum*. Prihantini and Tachibana^8^ showed *Pseudocercospora* sp. isolated from *Elaeocarpus sylvestris* could produce anti-oxidant compounds. Das et al.^9^ revealed the endophytic fungi isolated from *Zingiber nimmonii* produced caffeic acid, para-hydroxybenzoic acid, para-coumaric acid, vanillic acid, and syringic acid, which had anti-oxidant activities. In another study, Pan et al. showed that endophytic fungi isolated from Fritillaria unibracteata were capable of producing some phenolic acids such as caffeic acid, gallic acid, chlorogenic acid, ferulic acid and rosmarinic acid^10^. Mollaei et al.^4^ showed that *F. tricinctum*, as an endophytic fungus isolated from the roots of *Lithospermum officinale*, can be presented as a new source of shikonin. Hernández-Tasco et al.^11^ investigated the anti-oxidant activity of *Cladosporium* sp. isolated from *Annona cacans*, and succeed to identify compounds with anti-oxidant.

Fatty acids are other biologically active compounds that present many applications, due to their physical, biological, and alimentary properties. They were applied in the industries such as pharmaceuticals, cosmetics, and food. They adjust a wide range of functions in the human body, including regulation of inflammatory responses, brain maintenance and function, normal development, vision, vasodilation, vasoconstriction, and blood pressure^12–14^. Endophytic fungi isolated from plants are a common source for producing the fatty acids. Ghaffari et al.^4^ studied the fatty acid profile of endophytic fungi isolated from *Ziziphora tenuior*. They concluded that *Alternaria infectoria* and *Aspergillus ochraceus* had the highest amount of fatty acids, and oleic acid, stearic acid, palmitic acid, linoleic acid, and γ-linolenic acid were the main fatty acids. Yang et al.^15^ showed *Bionectria ochroleuca* isolated from *Torreya grandis* could produce some fatty acids such as siadonic acid, oleic acid, and linoleic acid.

Enzymes are biological catalysts that catalyze many chemical reactions and are applied in different industries such as detergent, pharmaceutical, food, etc. Due to the industrial use of enzymes, high-scale production with the highest level of activity and stability, as well as the lowest production cost, is very important. Based on the studies, endophytic fungi have the ability for producing different enzymes such as protease, cellulase, amylase, etc. Endophytic fungi such as *Fusarium* sp., *Aspergillus* sp., *Alternaria* sp., and *Penicillium* sp. were reported to produce extra cellular enzymes with a significant yield that can replace the prevailing resources^16^.

*Gundelia tournefortii* L. (belong to Asteraceae family) is an edible spiny, thistle-like plant which grows in the semi-desert areas of Asia. It's called tumble thistle in English and Kangar in Persian. The stem of *G. tournefortii* considered as a food and an herbal drug in Iran. In traditional medicine, *G. tournefortii* has been used as a hepatoprotective and blood purifier, and hypolipidemic and anti-oxidant agent, as well as anti-diabetes herb^17–20^. Since endophytic fungi isolated from plants have the ability to produce bioactive compounds, it is expected that *G. tournefortii* also hosts endophytic fungi that have the ability to biosynthesize active compounds. Therefore, the main goal of this study is to isolate and identify endophytic fungi from *G. tournefortii*, investigating their biological characteristics and studying their metabolites.

## Material and methods

### Isolation and molecular identification of endophytic fungi

Plant specimens of *G. tournefortii* were collected from Urmia, Iran (E45°04ʹ, N37°48ʹ), during May 2021. Voucher specimen after identifying by Dr. Mostafa Ebadi (ASMUH-40214) was deposited at the herbarium of Azarbaijan Shahid Madani University. Surface sterilization of the plant stems was done based on Sunitha et al.^21^ method, and inoculated into potato dextrose agar (PDA) to obtain pure colonies according to our previous study^6^. Total genomic DNA of was extracted by the Cetyltrimethylammonium bromide (CTAB) method.^22^ The fungal isolate was cultured on potato dextrose broth (PDB) and a small amount of the powdered mycelia was suspended in 10 ml of CTAB solution (50 mM Tris buffer pH 8.0, 100 mM EDTA, 150 mM NaCl) and incubated at 65 °C for 45 min. DNA was extracted by adding an equal volume of chloroform/isoamyl alcohol (24:1 v/v) and mixed thoroughly but gently and then centrifuged at 11,000 rpm for 15 min at 10 °C. Aqueous viscous supernatant was removed to a fresh tube and precipitated with 600 μl of ice-cold isopropanol and shaking for 5 min at 150 rpm. The mixture was centrifuged at 11,000 rpm for 10 min at 10 °C. Pellet was washed with 70% ethanol, dried completely, and dissolved in 100 μl amount of TE buffer. The extracted DNA was served as template for the amplification of ITS1-5.8S-ITS2 rRNA region. PCR amplification was performed by ITS 1 and ITS 4 as primers in a thermocycler (SensoQuest) programmed. The phylogenetic analyses were also constructed using the MEGA software ver. 6.0 with an alignment of the prepared sequences using the MUSCLE software. Finally, the phylogenetic tree was created using the neighbor-joining (NJ) algorithm with a p-distance substitution model and bootstrapping of 1000 replications^6^.

### Enzymatic activities of endophytic fungi

The amylase, cellulase, pectinase, and protease activates were performed by the Sunitha et al.^21^ method. For this propose, the endophytic fungi were cultivated on Yeast-Malt (YM) agar media supplemented with specific indicative substrates. The specific indicative substrates of amylase, cellulase, pectinase, and protease were 1% soluble starch, 1% carboxy methyl cellulose, 1% Pectin, and 1% gelatin, respectively. The enzyme production was determined based on the formation of a clear zone that appeared around the colonies of fungus.

### Extraction and analysis of fatty acids

To extract fatty acids, 0.5 g of dried and powdered endophyte fungi were poured into an Erlenmeyer flask and then n-hexane (50 mL) was added, and the extraction was done at ultrasonic bath for 30 min. Then, the sample was centrifuged for 5 min (5000 rpm). After collecting the upper phase, its solvent was evaporated by a rotary evaporator. The obtained extracts were weighed and the yield of the extract containing fatty acid was calculated.

The fatty acids were derivatized by alkaline method (REF), and analyzed by an YL 6100 GC gas chromatography device. The capillary column used (60 m × 0.25 mm × 0.2 µm) included 14% cyanopropyl phenyl-86% dimethyl polysiloxane, bonded and cross-linked phase. The initial temperature was 80 °C and it was raised to 120 °C (20 °C/min), and then reached 260 °C (3 °C/min), and was kept at this temperature for 10 min. The carrier gas was nitrogen and its speed was set at 1.1 mL/min. The amount of sample injection was 1 µL with a ratio of 1–20. The detector and injection temperatures were set at 280 and 260 °C, respectively. Fatty acids were identified using the standard and the percentage of each fatty acid was calculated using relative area percentages obtained by FID^23^.

### Total phenolic and flavonoid contents assay

To measure the Total Phenolic Content (TPC), the Folin-Ciocalteu method was used. Briefly, 20 µL of the extract (500 ppm) was added to 100 µL of Folin-Cicaltio reagent (10% v/v), and after 5 min, it was mixed with 20 µL of Na_2_CO_3_ (7% w/v). The samples were placed in the dark for 2 h. Finally, the absorbance of the samples was read by a spectrophotometer (765 nm). The TPC was determined as mg of gallic acid per equivalent gram of dry fungus (mgGAL/g) by the regression equation derived from the gallic acid standard curve^24^.

The Total Flavonoid Content (TFC) in the extracts was obtained by AlCl_3_ colorimetric method. Briefly, 20 µL of the extract (500 ppm) was added to 100 µL of aluminum chloride (3% w/v) and 0.1 mL of ammonium acetate (1 M). The obtained samples were kept at 25º for 10 min, and finally, the absorption of each sample was read at 426 nm. The TFC was calculated by the regression equation derived from the Quercetin, and was expressed as mg of quercetin per equivalent gram of dry fungi (mgQE/g)^24^.

### Extraction and analysis of phenolic acids

To extraction phenolic acids, 2 ml of Merck's methanol solvent containing 0.1% HCl (1 M) was added to 0.2 g of defatted fungi samples and placed inside the ultrasonic device at 25 °C. After 30 min, the extract was filtered and its solvent was evaporated. Water (2 mL) was added to the sample, and was mixed with n-hexane (1:1 v/v). The obtained sample was shaken for two min, and the aqueous phase was separated and mixed with diethyl ether and ethyl acetate solvents (1:1 v/v). After shaking for 5 min, the organic phase was separated and evaporated. Finally, the extract was placed inside the oven at 60 °C to dry completely.

The separation and identification of phenolic acids was done by HPLC^25^. The column used was C18 (250 mm × 0.46 mm, 5 μm). Also, the mobile phase was HPLC grade methanol and water with 0.1% acid with a flow rate of 0.5 mL/min and a gradient washing system. The injection volume was 20 µL and the type of detector used was UV, which was set at a wavelength of 275 nm. The amount of phenolic acids was calculated by the regression equation derived from standards, and was expressed as mg per gram of dry fungi (mg/g).

### Anti-oxidant activity by DPPH method

The anti-oxidant activity was evaluated by measuring the amount of free radicals DPPH^26^. Briefly, 100 µL of the extract with various concentrations (0.2, 0.4, 0.7, 1.0, and 1.25 mg/mL) was added to DPPH solution (100 µL, 0.1 mM), and mixed for 30 min at 25 °C. Then, the absorbance of the samples was read at 517 nm. The inhibition percentage of DPPH free radicals was determined, and finally, the IC_50_ value of the extract was calculated. In this study, ascorbic acid was used as positive control, and methanol as negative control.

### Statistical analysis

Each experiment was repeated at three times, and data were analyzed using the SPSS ver.17 using one-way analysis of variance (ANOVA) of a completely randomized design, and the mean comparisons were determined by Tukey's test (*p* < 0.05).

## Results

### Fungi isolation

**Figure 1:**
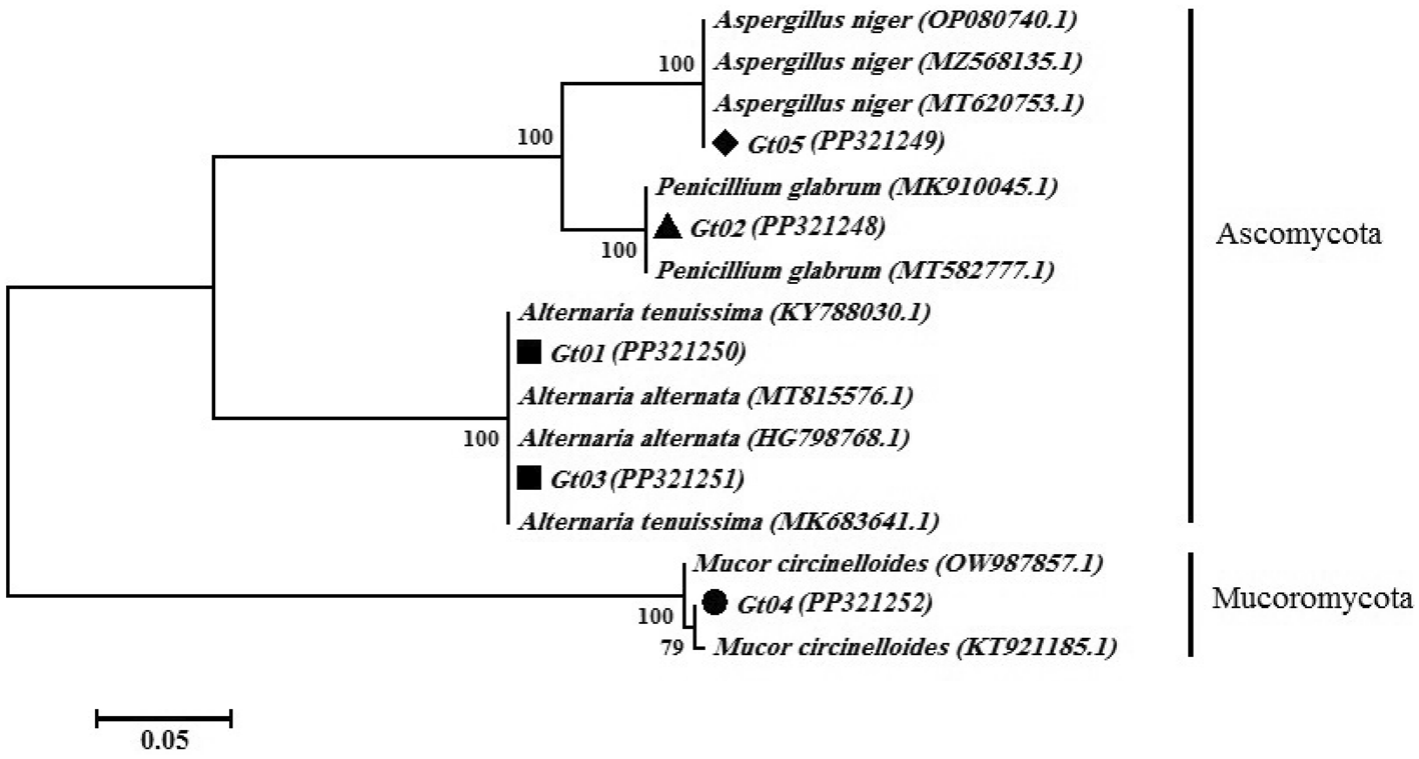
Phylogenetic analysis of endophytic fungi isolated from *G*. *tournefortii* based on the internal transcribed spacer. The phylogenetic tree was constructed using the neighbor-joining method (1000 bootstrap replications).

The results revealed that *G. tournefortii* associated with some endophytic fungi. Totally, five isolates (Gt01-05) were obtained from the *G. tournefortii* stems. According to the phylogenetic analysis, the identified species are placed in three family including Aspergillaceae, Pleosporaceae, and Mucoraceae (Fig. 1). The first clade contained the isolates grouped with 100% bootstrapping to *Aspergillus niger* (Gt05) and *Penicillium glabrum* (Gt02) sequences. The second clade was grouped to *Alternaria alternata* and *A. tenuissima* sequences (Gt01 and Gt03) retrieved from NCBI with 100% bootstrapping. The third clade comprised the isolate (Gt04) grouped with 100% bootstrapping to *Mucor circinelloides* sequences obtained from NCBI (Fig. 1).

### Enzyme assay

Evaluation of the enzymatic properties of endophytic fungal isolates showed that *P. glabrum* and *A. niger* (Gt02 and Gt05) had the greatest ability for producing all studied extracellular enzymes (Table 1). *A. alternata* and *M. circinelloides* (Gt01 and Gt04) showed extracellular property for amylase and pectinase; while, they indicated negative extracellular property for cellulase and protease. *A. tenuissima* (Gt03) showed extracellular property for amylase, cellulase, and pectinase. Although maximum extracellular amylase property was obtained in *A. alternata* followed by *P. glabrum* isolate, ANOVA analysis indicated that variations between these isolates to carboxymethyl cellulose (CMC) substrates did not reach significant level (*P* ≤ 0.05).

**Table 1:** Extracellular enzymatic activities of fungal endophytes using solid media.

Fungi isolate	Diameter of clear zones (mm)			
	Amylase	Cellulase	Pectinase	Protease
*A. alternata* (Gt 01)	41 ± 1.3^a^	0^a^	10 ± 0.5^a^	0^a^
*P. glabrum* (Gt02)	39 ± 1.5^ab^	15 ± 1.2^b^	31 ± 1.9^b^	12 ± 0.9^b^
*A. tenuissima* (Gt03)	32 ± 0.8^c^	4 ± 0.5^c^	17 ± 1.5^c^	0^a^
*M. circinelloides* (Gt04)	37 ± 2.1^b^	0^a^	24 ± 0.6^d^	0^a^
*A. niger* (Gt05)	5 ± 0.3^d^	23 ± 1.7^d^	22 ± 1.8^d^	9 ± 1^c^
Control	0^e^	0^a^	0^e^	0^a^

In a column, values are the means ± SE followed by different letters are significantly different (*P * < 0.05) by Tukey test (n = 3).

Among the studied endophytes, three isolates could produce cellulase enzymes and developed a clear zone on the CMC medium. A significant difference in cellulase activity was detected between tested isolates and maximum activity was recorded in *A. alternata* (Table 1).

The difference between pectinase properties of *M. circinelloides* and *A. niger* did not reach significant level. But, pectinase activity of *P. glabrum* was significantly higher as compared to other tested entophytes (Table 1).

Among the tested endophytes, *P. glabrum* and *A. niger* showed protease activity, whereas *P. glabrum* significantly exhibited high proteolytic property compared to *A. niger* (P ≤ 0.05) (Table 1).

### Investigation of anti-oxidant property

The results of anti-oxidant property showed that the inhibition of free radicals depended on the concentration of the extracts, and with the increase of their concentration, the rate of inhibition of free radicals increased. In the concentration range, ascorbic acid (synthetic anti-oxidant) had a high anti-oxidant activity compared to all samples, and in this sense, none of the samples had the ability to compete with ascorbic acid.

**Figure 2:**
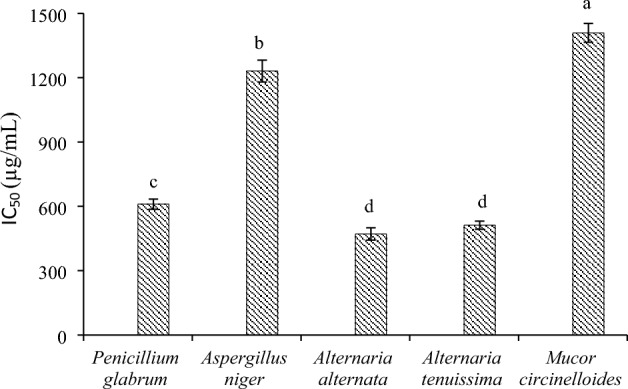
Anti-oxidant activity of the extract of endophytic fungi isolated from *Gundelia tournefortii.* The experiment was performed in three repetitions (mean ± SD), and different uppercase letters indicate statistically significant difference (*p* < 0.05).

Figure 2 shows the IC_50_ value of extracts. Based on the results, *A. alternata* extract (IC_50_ = 471 ± 29 µg/mL) had the highest anti-oxidant properties, followed by *A. tenuissima* extract (IC_50_ = 512 ± 29 µg/mL). It should be explained that compared to ascorbic acid (IC_50_ = 82 ± 3 μg/mL), the extracts did not had good anti-oxidant properties. *M. circinelloides* and *A. niger* extracts also had no anti-oxidant properties. According to the obtained results, it could be assumed that the high anti-oxidant properties of *A. alternata* and *A. tenuissima* extracts compared to other extracts could be related to their phenolic and flavonoids compounds, and their total phenol and flavonoid contents could justify their high anti-oxidant activity.

### Analysis of phenolic compounds

**Figure 3:**
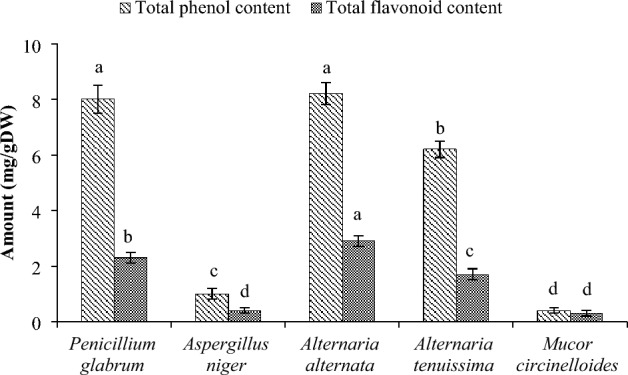
Total phenol and flavonoid contents in endophytic fungi isolated from *Gundelia tournefortii.* Data were calculated as the mean ± SD of three measurements and represented along with the error bar. In each parameter, different uppercase letters indicate statistically significant difference (*p* < 0.05).

The polyphenolic extracts of fungi were evaluated in terms of total phenol and flavonoid content. As can be seen from Fig. 3, the extracts of *P. glabrum* and *A. alternata* had the highest amount of total phenols (8.2 ± 0.4 and 8.0 ± 0.5 mgGAL/gDW, respectively). The TFC results indicated that the *A. alternata* extract had the highest amount of total flavonoids with 2.9 ± 0.3 mgQE/g. The *P. glabrum* extract also ranked second with 2.3 ± 0.2 mgQE/g. Also, the lowest amount of total flavonoid was related to the *M. circinelloides* and *A. niger* extracts.

**Figure 4:**
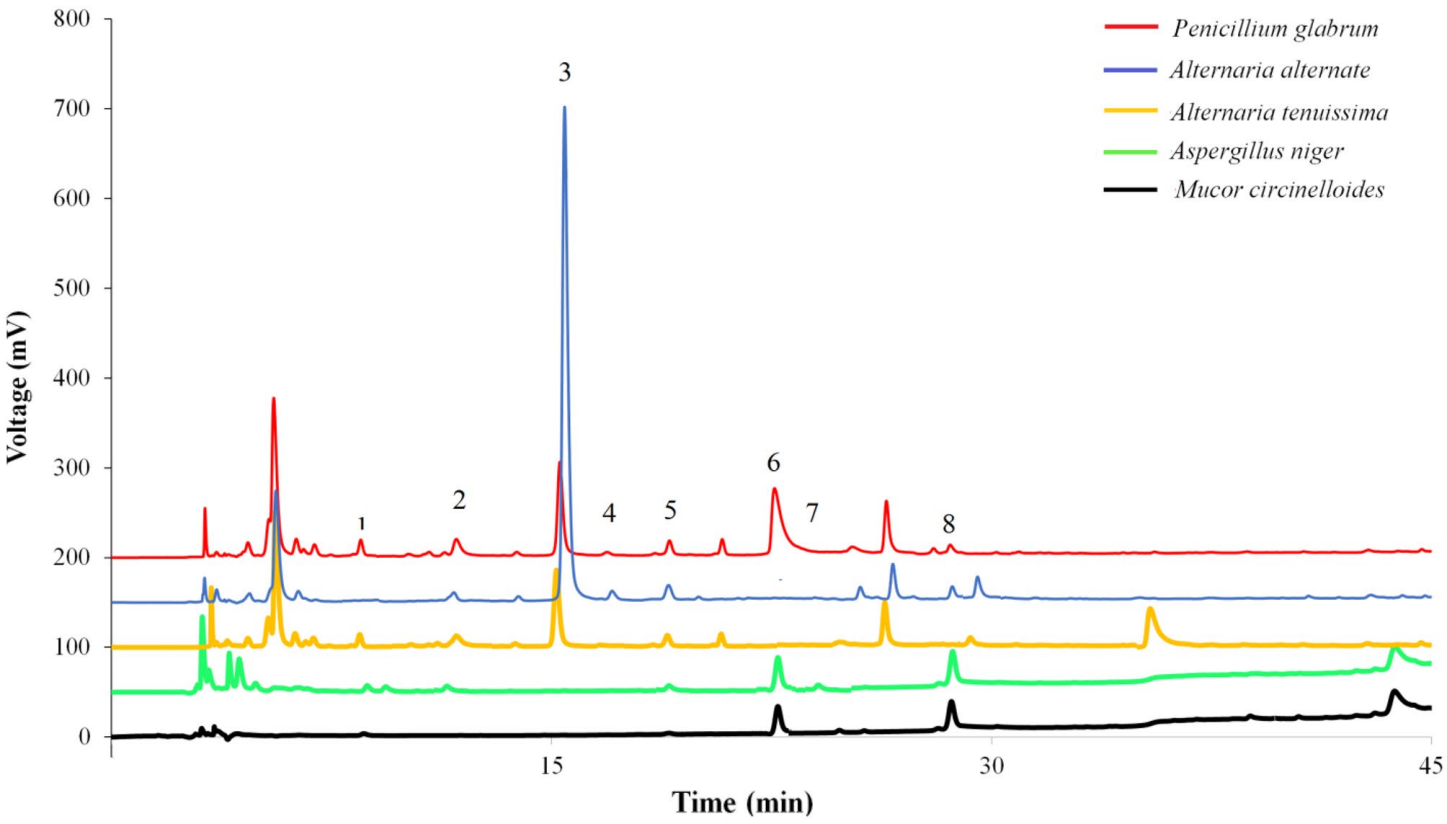
HPLC chromatograms of the phenolic extracts obtained from endophytic fungi. The column was C18 (250 mm × 0.46 mm, 5 μm), and signal was recorded at 254 nm; Peak assignments: 1- gallic acid, 2- protocatechuic acid, 3- rosmarinic acid, 4- p-hydroxybenzoic acid, 5- caffeic acid, 6- p-coumaric acid, 7- ferulic acid, and 8- cinnamic acid, respectively.

The phenolic acids profiles of the endophytic fungi extracts were investigated using HPLC. HPLC chromatograms related to the extracts were shown at Fig. 4. Table 2 showed the phenolic acids compounds in the endophytic fungi extract. Based on the results, caffeic acid was the only phenolic acid that was detected in all the extracts. The *A. tenuissima* extract had the highest amount of rosmarinic acid with the amount of 42.02 ± 1.31 mg/g dry weight of the fungi (mg/g DW), followed by the *P. glabrum* and *A. alternata* extracts. The second major acid present in the extracts was related to para-coumaric acid, and the *P. glabrum* extract had the highest amount of this compound with the amount of 7.53 ± 0.19 mg/g DW. meta-Coumaric acid was another compound that had the highest amount in the extracts of *A. niger* and *M. circinelloides*, and its amounts were 5.41 ± 0.41 and 4.37 ± 0.12 mg/g DW, respectively. Among the studied phenolic acids, vanillic acid, salicylic acid and cinnamic acid compounds were not detected in any of the extracts. Ferulic acid was also present only in the *A. niger* extract (0.27 ± 0.02 mg/g DW). Gallic acid was present in all extracts except *A. tenuissima* extract. Procatechuic acid was also not found only in the *M. circinelloides* extract.

**Table 2:** Amount of phenolic acid compounds (mg/kg dried plant) in fungal isolates.

Phenolic acids	*Penicillium glabrum*	*Aspergillus niger*	*Alternaria tenuissima*	*Alternaria alternata*	*Mucor circinelloides*
Gallic acid	0.15 ± 0.01^d^	0.03 ± 0.00^c^	0.14 ± 0.02^c^	0.00	0.02 ± 0.00^c^
Protocatechuic acid	0.18 ± 0.01^d^	0.08 ± 0.01^c^	0.16 ± 0.01^c^	0.15 ± 0.01^d^	0.00
Rosmarinic acid	4.02 ± 0.16^b^	0.00	3.23 ± 0.12^a^	41.02 ± 1.31^a^	0.00
p-Hydroxybenzoic acid	0.01 ± 0.00	0.00	0.00	0.07 ± 0.00 e	0.00
Vanillic acid	0.00	0.00	0.00	0.00	0.00
Caffeic acid	0.15 ± 0.01^d^	0.05 ± 0.00^c^	0.22 ± 0.03^b^	0.32 ± 0.02^c^	0.04
p-Coumaric acid	7.53 ± 0.19^a^	0.23 ± 0.02^b^	0.00	0.00	0.32 ± 0.02^b^
Ferulic acid	0.00	0.27 ± 0.01^b^	0.00	0.00	0.00
m-Coumaric acid	0.43 ± 0.03^c^	5.41 ± 0.21^a^	0.00	0.51 ± 0.05^b^	4.37 ± 0.12^a^
Salicylic acid	0.00	0.00	0.00	0.00	0.00
Cinnamic acid	0.00	0.00	0.00	0.00	0.00

In a column, values are the means ± SE followed by different letters are significantly different (*P *< 0.05) by Tukey test (n = 3).

### Analysis of fatty acids

**Figure 5:**
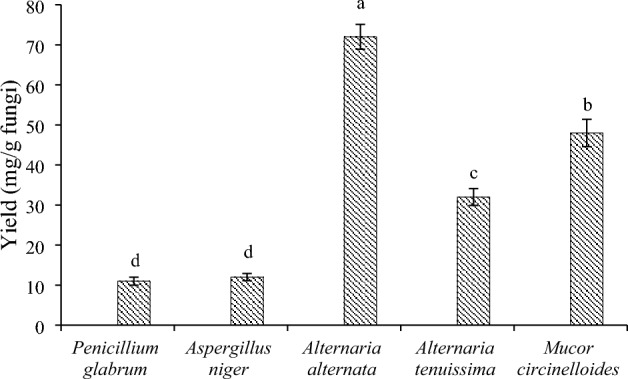
Hexane extract yield of endophytic fungi isolated from *Gundelia tournefortii.* The experiment was performed in three repetitions and the columns with the same letters had no significant difference at the 5% probability level.

Figure 5 shows the yield of hexane extract of endophyte fungi. Based on the obtained results, *A. alternata* extract followed by *M. circinelloides* had the highest yield with values of 72.0 ± 3.1 and 48.0 ± 3.4 mg/g DW, respectively. *P. glabrum* and *A. niger* had the lowest yield (11.0 ± 1.0 and 12.0 ± 0.9 mg/g, respectively).

To investigate the profile of fatty acids, the n-hexane extracts were analyzed by a gas chromatography device. Based on the results (Table 3), twenty fatty acids were identified and the fatty acid percentage of the extracts was reported between 96.9 and 99.0% (Table 3). In all fungi, the main fatty acids were stearic acid (27.9–35.2%), oleic acid (11.3–17.3%), palmitic acid (16.9–23.2%), linoleic acid (5.8–11.6%), and caprylic acid (6.3–10.9%). Also, Fatty acids were grouped according to the availability of saturated, monounsaturated, and polyunsaturated fatty acids. The results showed that saturated fatty acid was the major group.

**Table 3:** Fatty acid composition (%) of fungal isolates.

Fatty acid	Common name	*Penicillium glabrum*	*Aspergillus niger*	*Alternaria tenuissima*	*Alternaria alternata*	*Mucor circinelloides*
C8:0	Caprylic acid	6.3	10.6	6.8	6.3	10.9
C10:0	Capric acid	1.3	4.2	2.0	4.3	4.5
C12:0	Lauric acid	0.5	1.0	1.0	1.0	2.8
C14:0	Myristic acid	1.3	1.1	0.9	0.9	2.2
C14:1	Myristoleic acid	1.1	0.9	1.6	1.3	1.7
‍‍‍C15:0	Pentadecylic acid	1.7	1.7	1.8	1.4	1.9
‍‍‍C15:1	Pentadecenoic acid	1.3	1.0	1.5	1.4	1.2
C16:0	Palmitic acid	18.7	19.8	21.7	23.2	16.9
C16:1	Palmitoleic acid	2.3	0.7	2.3	1.1	2.6
C18:0	Stearic acid	27.9	34.5	30.8	35.2	28.1
C18:1(n-9)	Oleic acid	17.3	10.3	15.3	10.9	13.2
C18:2(n-6)	Linoleic acid	11.6	5.8	6.6	6.2	6.3
C18:3 n3	α-Linolenic acid	2.1	1.7	1.7	0.7	1.5
C20:0	Eicosanoic acid	1.1	1.1	1.0	1.1	1.3
C20:2	Eicosadienoic acid	1.2	0.8	1.1	0.9	0.6
C20:3 n3	Eicosatrienoic acid	1.3	1.0	1.1	0.4	1.0
C20:4 n6	Arachidonic acid	0.0	0.5	0.8	0.3	0.6
C20:5 n3	Eicosapentaenoic acid	0.4	0.5	0.2	0.3	0.3
C22:4 n6	Docosatetraenoic acid	0.0	0.6	0.7	0.0	0.3
C22:5 n3	Docosapentaenoic acid	1.6	0.0	0.0	0.0	0.0
SFA		58.8	74	66	73.4	68.6
MUFA		22	12.9	20.7	14.7	18.7
PUFA		18.2	11.1	12.2	8.8	10.6
SUM		99.0	97.8	98.9	96.9	97.9

## Discussion

### Fungi isolation

The use of molecular phylogeny is very necessary and useful for the accurate identification of fungal species. In this research, according to the results of phylogenetic analysis, the studied isolates were placed in separate clades and their taxonomic position was possible based on the ITS region. The isolates Gt01 and Gt02, which belonged to *A. alternata* and *A. tenuissima*, were placed in the same clade and, although the ITS region was able to show the taxonomic position of these isolates, but it was not able to separate the two species. Similar to this finding, Zhao et al.^27^ showed that it is not possible to separate between *A. alternata* and *A. tenuissima* based on the ITS region, and the examination of morphological traits is necessary. The color of the dorsal surface of *A. alternata* and *A. tenuissima* species in the PDA is olive green and gray to dark brown, respectively. Also, the conidial chain of *A. alternata* has two to three lateral branches, while the conidial chain of *A. tenuissima* has one lateral branch. According to morphological characteristics, isolate Gt01 belongs to *A. tenuissima* and isolate Gt03 belongs to *A. alternata*.

### Enzyme assay

Five fungal endophytes were isolated from *G. tournefortii*, a medicinal native plant species in Iran, that they have various biological and biochemical properties potentially useful. The results showed that the studied isolates had ability for producing different enzymes regarding to degrade starch, carboxy methyl cellulose, pectin, and gelatine. *P. gabrum* and *A. niger* had high ability for producing most of the studied extracellular enzymes, unlike to *A. alternata* and *M*. *circinelloides* which only was able to degrade starch and pectin. Sunitha et al.^21^ and García-Latorre et al.^28^ showed high property of extracellular enzymes in fungal endophytes associated with plants. In association with their host plants, endophytic fungi secrete proteins presumed to aid in growth, nourishment, and defense. The endophytic fungi produce some enzymes such as lipases, proteases, laccases, amylases, cellulases, and pectinases as one of the resistant mechanisms against pathogenic organisms and also for gaining nutrients from the host. The cellulose and lignin production shows a strategic benefit to decompose the tissues and endure as saprobes after senescence^29^. Our results supported that enzymatic property of endophytes plays main role for degradation of protein and polysaccharides, which could imply a resistance strategy of the host plant against pathogenic infection^29^.

### Anti-oxidant phenolic compounds from endophytic fungi

Free radicals are compounds with single electrons that irreversibly react with biological molecules such as lipoproteins, lipids, carbohydrates, nucleic acids, proteins and free amino acids. They damage and cause many diseases such as cancer, cataracts, cardiovascular diseases, etc. The anti-oxidant defense systems include enzymatic and non-enzymatic factors. Enzymatic factors such as glutathione peroxidase, superoxide dismutase and catalase inhibit peroxide, superoxide and hydrogen peroxide radicals, respectively. Non-enzymatic factors such as phenols inhibit many reactive oxygen species and free radicals. These compounds are valuable secondary metabolites that are abundant in some plants and have many biological benefits. They are classified into different groups such as tannins, stilbenes, lignins, lignans, coumarins, quinones, phenolic acids and flavonoids, and endophytic fungi can be an important source for the production of these compounds^30^.

In this study, it was observed that *Alternaria* sp. had the highest amount of phenolic compounds. Also, based on the IC_50_ results, these fungi (i.e. *A. alternata* and *A. tenuissima*) had the highest anti-oxidant properties. It can be assumed that the high anti-oxidant properties of these extracts can be related to their phenolic and flavonoids compounds. Up to now, some studied have been done on the phenolic compounds and anti-oxidant properties of *Alternaria* sp. Elghaffar et al.^31^ evaluated the anti-oxidant property of the *A. alternata* extract which was isolated from the *Ziziphus spina-christi* leaves. The results revealed that this extract with an IC_50_ value of 409 μg/mL had an acceptable anti-oxidant property, and this property was attributed to the presence of phenolic compounds. Elbermawi et al.^32^ identified six phenolic compounds in *Alternaria* sp. isolated from fresh leaves of *Lycium schweinfurthii*, *Pancratium maritimum* and *Cynanchum acutum*. These compounds were named alternariol-5-O-methyl ether, alternariol, altenusin, altenuene, alternarienoic acid, and talaroflavone. Examining their biological properties showed that some of these compounds had strong alpha-glucosidase and lipase inhibitory activities and may act as natural anti-diabetic candidates. Zheng et al.^33^ succeeded in isolating eight endophytic fungi that produce flavonoid compounds from the branches of *Loranthus tanakae* and concluded that *Alternaria tenuissima* ZP28 and ZM148 have better anti-oxidant activity than others. Then, its flavonoid compounds were identified by LC–MS. Ibrahim et al.^34^ indicated that *Alternaria* species isolated from *Dracaena* had a potential anti-oxidant activity, and its IC_50_ was 520 μg/mL. In another study, 28 isolates of endophytic fungi including *Plectosphaerella*, *Alternaria, Aspergillus*, and *Fusarium* were isolated from *Ferula assa-foetida*, and their ability to biosynthesis of phenolic acids was studied. The highest amount of total flavonoid, total phenol, and anti-oxidant activity was reported in *A. tenuissima* supernatant. The identification of 14 phenolic acids showed that rosmarinic acid (64.11 mg/g DW) was major phenolic acid. Finally, *A. tenuissima* was introduced as a rich source of rosmarinic acid^5^. *A. alternata* isolated from *Azadirachta indica* has the ability to produce compounds with strong anti-bacterial and anti-oxidant properties, and the investigation on these compounds showed that they belong to flavonoids^35^. Gunasekaran et al.^36^ concluded that the *Alternaria* sp. extract inhibited DPPH free radicals up to 85.20% at a concentration of 300 μg/mL. In another study, the separation of *Alternaria* sp. Samif01 isolated from *Salvia miltiorrhiza* led to the isolation of seven phenolic compounds belonging to dibenzo-α-pyrones. Examining the anti-bacterial properties of these compounds showed that some of these compounds had inhibitory properties against Gram-negative and Gram-positive bacteria in the range of 86–364 µM. Moreover, these compounds showed high anti-oxidant and cytotoxic activities^37^. In 2015, Anyanwu and Sorensen^38^ succeeded in isolating two phenolic compounds named Deoxyphomalone and stemphyperylenol from *A. tenuissima*. Fang et al.^39^ identified several phenolic compounds from *A. tenuissima* isolated from *Erythrophleum fordii*. They included α-Acetylorcinol (belonging to phenylpropenes) and tenuissimasatin (belonging to isocoumarins). Further, they showed that these compounds had cytotoxic properties against human colon cancer cells. Therefore, there was almost a significant relationship between the anti-oxidant properties of the endophytic fungi studied in this research with previous studies.

According to phenolic content and anti-oxidant properties of *Alternaria* sp. extracts, their phenolic acids were studied using HPLC. The results indicated that rosmarinic acid was the main phenolic acid in the *A. alternata*. This compound is an ester of caffeic acid, which is famous as a phenolic compound. This compound was reported for the first time from the rosemary plant, and then was reported from some other natural sources such as plants and endophyte fungi^40^. In a research done by Parvandi et al.^5^, 28 isolates of endophytic fungi were isolated from *Ferula assa-foetida*. Then, their abilities for producing phenolic acids were studied, and it was found that *Alternaria* sp. was a rich source of rosmarinic acid. Therefore, this research along with our results showed that *A. alternata* could be a source of rosmarinic acid. Since this compound has many biological properties such as anti-viral, anti-bacterial, anti-cancer, anti-oxidant, anti-aging, anti-diabetic, heart protection, liver protection, anti-depressant, anti-allergy and anti-inflammatory activities, and also, since ancient times, it has been used in folk medicine, cosmetics and food supplements^40^.

*P. glabrum* was another endophytic fungi which anti-oxidant activity. *Penicillium* species have the potential to produce various phenolic compounds with high biological properties^41^. Some anti-oxidant phenolic compounds were extracted from *Penicillium* spp*.*, and revealed that these compounds could be applied at food industries^42^. Jakovljević et al.^43^ investigated the chemical compounds and biological activities of *Penicillium* spp. In this study, anti-oxidant activity was investigated by various methods. The results showed that *P. chrysogenum* had higher TPC, and better anti-oxidant property, and also had the ability to chelate iron ions and could be a new potential source of natural anti-oxidants. According to our results, para-coumaric acid was the major phenolic acid in its extract. para-Coumaric acid is a phenolic acid found in fruits, vegetables and fungi. This compound can be converted into other phenolic acids such as sinapic acid, caffeic acid, and ferulic acid, also other metabolite acids such as lignins and lignin precursors. The studies have shown that para-coumaric acid has several biological activities such as anti-inflammatory, anti-oxidant, anti-platelet aggregation, analgesic, anti-cancer and neuroprotective^44,45^. According to our results, since *P. glabrum* had the ability to produce para-coumaric acid, it could be considered as a suitable source for the production of this compound on a commercial scale.

As a result, from our results and previous studies, it could be concluded that the anti-oxidant properties of *Alternaria* sp. and *P. glabrum* may be due to the presence of phenolic acids compounds such as rosmarinic acid and para-coumaric acid.

### Analysis of fatty acids

To investigate the profile of fatty acids, the n-hexane extracts were analyzed, and the results indicated that stearic acid, oleic acid, and linoleic acid were the main fatty acids in studied fungi. Based on the previous studies, these compounds have many properties and are used in different industries.

Stearic acid is a chemical compound that exists naturally in some plants and fungi. This compound is known as a strong organic acid and is used in many chemical, pharmaceutical and agricultural industries. This compound was used as an anti-bacterial and anti-fungal agent. Also, this compound is used in the production of detergents, paints, plastics and other industries. Other uses of stearic acid include pH control in various industries, production of alkaline batteries, production of organic fertilizers and production of food preservatives^46,47^.

Oleic acid is an unsaturated acid that is found in abundance in nature, is widely used in the industry. Also, it is used in cosmetics and health sectors such as making softening lotions, soaps, etc. This compound decreases blood cholesterol and prevents it from increasing again, regulates blood sugar, strengthens the defense and immunity system of the body, and keeps the skin moist and prevents premature aging. Also, it is one of the important substances to destroy cancer cells (breast cancer in women). In diet programs, the use of this substance causes weight loss^47,48^.

Linoleic acid is a nutrient that has two double bonds. It is one of the unsaturated and essential fatty acid that is often found in vegetable oils. This fatty acid is also known as omega-6 fatty acid. It is very essential for human nutrition because its synthesis process cannot be done by the body. Linoleic acid greatly reduces the risk of cardiovascular diseases, promotes healthy skin and hair, improves the health of the brain, strengthens the body's immune function, protects bone density, reduces inflammation in joints, digestive system, lungs and transplanted brain, etc^49,50^.

## Conclusion

In this study, the molecular identification, extracellular enzymetic activity as well as phenolic and fatty acid profile analysis, and anti-oxidant properties of endophytic fungi isolated from the stems of *G. tournefortii* were investigated. According to phylogenetic analysis five isolates were identified as following: *A. niger*, *P. glabrum*, *A. alternata*, *A. tenuissima*, and *M. circinelloides*. Evaluation of the enzymatic properties showed that *P. gabrum* and *A. niger* had more ability for producing most of the studied extracellular enzymes. The anti-oxidant activity showed that *A. alternata* extract had the highest anti-oxidant properties, total phenols and flavonoids contents. The quantification analysis of metabolites indicated that rosmarinic acid and stearic acid were the main compounds in studied fungi. In general, the results of this study showed that endophytic fungi have a high potential for the producing biologically active substances, and due to the quick and easy cultivation of these fungi, the production of biologically active substances is valuable. In the future studies, approaches to increase metabolites and enzyme activities by optimizing growth parameters, co-cultivation, and overexpression of genes can be investigated. Also, an attempt can be made to the application of endophytic fungi in agriculture, biofuel, therapeutics, the food industry, and other relevant industries.
